# Are There Mental Health Differences Between Francophone and Non-Francophone Populations in Manitoba?

**DOI:** 10.1177/070674371405900704

**Published:** 2014-07

**Authors:** Mariette Jeanne Chartier, Gregory Finlayson, Heather Prior, Kari-Lynne McGowan, Hui Chen, Randy Walld, Janelle de Rocquigny

**Affiliations:** 1 Research Scientist, Manitoba Centre for Health Policy, Department of Community Health Sciences, Faculty of Medicine, University of Manitoba, Winnipeg, Manitoba; Assistant Professor, Department of Community Health Sciences, Faculty of Medicine, University of Manitoba, Winnipeg, Manitoba.; 2 Research Scientist, Manitoba Centre for Health Policy, Department of Community Health Sciences, Faculty of Medicine, University of Manitoba, Winnipeg, Manitoba.; 3 Data Analyst, Manitoba Centre for Health Policy, Department of Community Health Sciences, Faculty of Medicine, University of Manitoba, Winnipeg, Manitoba.; 4 Research Coordinator, Manitoba Centre for Health Policy, Department of Community Health Sciences, Faculty of Medicine, University of Manitoba, Winnipeg, Manitoba.; 5 Graduate Student, Department of Community Health Sciences, Faculty of Medicine, University of Manitoba, Winnipeg, Manitoba.

**Keywords:** mental health, psychiatric services, minority, Francophone, Manitoba, promotion, language barriers, cultural barriers

## Abstract

**Objective:**

Francophones may experience poorer health due to social status, cultural differences in lifestyle and attitudes, and language barriers to health care. Our study sought to compare mental health indicators between Francophones and non-Francophones living in the province of Manitoba.

**Methods:**

Two populations were used: one from administrative datasets housed at the Manitoba Centre for Health Policy and the other from representative survey samples. The administrative datasets contained data from physician billings, hospitalizations, prescription drug use, education, and social services use, and surveys included indicators on language variables and on self-rated health.

**Results:**

Outside urban areas, Francophones had lower rates of diagnosed substance use disorder (rate ratio [RR] = 0.80; 95% CI 0.68 to 0.95) and of suicide and suicide attempts (RR = 0.59; 95% CI 0.43 to 0.79), compared with non-Francophones, but no differences were found between the groups across the province in rates of diagnosed mood disorders, anxiety disorders, dementia, or any mental disorders after adjusting for age, sex, and geographic area. When surveyed, Francophones were less likely than non-Francophones to report that their mental health was excellent, very good, or good (66.9%, compared with 74.2%).

**Conclusions:**

The discrepancy in how Francophones view their mental health and their rates of diagnosed mental disorders may be related to health seeking behaviours in the Francophone population. Community and government agencies should try to improve the mental health of this population through mental health promotion and by addressing language and cultural barriers to health services.

## Abstract (fr)

**Objectif:**

Les francophones peuvent avoir une moins bonne santé en raison du statut social, des différences culturelles de style de vie et de mentalité, et des obstacles linguistiques aux soins de santé. Notre étude cherchait à comparer les indicateurs de santé mentale entre francophones et non-francophones habitant dans la province du Manitoba.

**Méthodes:**

Deux populations ont été utilisées : l’une tirée des bases de données administratives du Centre des politiques de santé du Manitoba et l’autre, issue d’échantillons d’enquête représentatifs. Les bases de données administratives contenaient des données de facturation des médecins, d’hospitalisations, d’utilisation de médicaments sur ordonnance, d’éducation, et d’utilisation des services sociaux, alors que les enquêtes incluaient des indicateurs des variables linguistiques et de la santé autoévaluée.

**Résultats:**

Hors des régions urbaines, les francophones avaient des taux plus faibles de trouble lié à une substance diagnostiqué (rapport de taux [RT] = 0,80; IC à 95 % 0,68 à 0,95) et de suicide ou de tentatives de suicide (RT = 0,59; IC à 95 % 0,43 à 0,79), comparativement aux non-francophones, mais aucune différence n’a été observée entre les groupes à l’échelle de la province en ce qui concerne les taux de troubles de l’humeur, de troubles anxieux, et de démence diagnostiqués, ou de tout trouble mental après correction pour l’âge, le sexe, et la région géographique. Lorsqu’interrogés, les francophones étaient moins susceptibles que les non-francophones de déclarer que leur santé mentale était excellente, très bonne, ou bonne (66,9 %, comparé à 74,2 %).

**Conclusions:**

L’écart entre la façon dont les francophones voient leur santé mentale et leurs taux de troubles mentaux diagnostiqués peut être lié aux comportements favorisant la santé dans la population francophone. Les organismes communautaires et gouvernementaux devraient tenter d’améliorer la santé mentale de cette population en faisant la promotion de la santé mentale et en réduisant les obstacles linguistiques et culturels aux services de santé.

Mental health has profound effects on the overall well-being and functioning of people, their families, and their communities. Mental health problems negatively impact quality of life,^1^ disability leave from work,^2^ and after musculoskeletal and cardiovascular diseases, are estimated to be the most costly health problems to society.^3^ Our study sought to gain a better understanding of the mental health of Francophones living in the province of Manitoba. Some previous studies suggest that Francophones living in Canada may have poorer mental health, compared with other Canadians^4^,^5^; however, these findings are not consistent across studies. The findings differ by mental health indicators, by province, by time period, and by methodology, leaving it unclear whether the mental health of this linguistic group is poorer than that of the general population. Differences may exist in health status of Francophones across Canada owing to differences in social status, cultural differences in lifestyle and attitudes, or in language or cultural barriers related to health care.^6^ More recent studies with the CCHS data found that once adjustments were made for numerous sociodemographic, economic, and cultural factors, Francophones were equally likely to report the presence of a mental disorder within the last year as other linguistic groups.^7^

Our study sought to compare the rates of mental health indicators of Francophones and non-Francophones in 2 study populations. Based on previous studies, we hypothesize that the mental health of Francophones in Manitoba will not be different if their SES is comparable with that of non-Francophones. Extensive data linkage using administrative and survey databases was required to create the matched cohort by selecting Francophones and non-Francophones while maintaining their anonymity. Having better knowledge of the mental health status of a population is helpful for policy development and service planning and contributes to the vitality of that population.

## Methods

### Study Population

We examined 2 study populations: one from the Population Health Research Data Repository (referred to henceforth as the Repository) housed at the MCHP at the University of Manitoba, and the other from representative surveys, namely, the CCHS, the NPHS, and the Manitoba HHS. Both study populations are from the province of Manitoba, located in the centre of Canada. Manitoba has a population of 1.2 million, with more than one-half of the population living in the provincial capital city of Winnipeg. The other half of the population lives in rural and northern regions or in small cities with populations of 60 000 or less.

**Clinical Implications**

- Francophones living in areas where they are a minority may not be comfortable seeking help for mental health concerns.
- Offering French-language health services may improve access to mental health services for Francophones.

**Limitations**

- Mental disorder indicators were from administrative data and miss people who are not seeking help for mental health concerns.
- A selection bias may be present as not all Francophones were identified with the use of administrative databases.

A total of 40 600 Francophones and 121 800 non-Francophones were included in the Francophone and matched cohort using the Repository databases at the MCHP. These databases were scanned for language flags, such as maternal languages, linguistic preferences, or self-identification as a Francophone. The Repository houses a collection of administrative databases that were originally collected to administer health, education, and social services in Manitoba. These data capture virtually all contacts by Manitoba residents when they access provincial services. The Repository holds no personal identifying information, such as names and addresses; and a numeric identifier is scrambled before the data are deposited in the Repository. These datasets are linkable at an individual level across files and over time because the identifier is scrambled in the same way for each file. The data in the Repository have been examined extensively and validated for research purposes.^8^–^14^ Our study received approval from the Health Research Ethics Board at the University of Manitoba and the Health Information Privacy Committee of Manitoba. Written consents were not required as we used de-identified administrative data.

Several steps were required to create the Francophone cohort, including the selection of Francophones using MCHP administrative data and survey data, as well as the addition of first-degree family members through health registry linkage. An initial cohort of 19 396 Francophones was created from the administrative and survey databases using language flags. These language indicators varied across databases in both how they were recorded and how well they were captured. They included attending a school offering a Français program, residence in Francophone personal care homes, attending a Francophone child care centre, requesting health-related correspondence in French in the home care or the immunization programs, as well as having French as their maternal language on a kindergarten developmental measure, and reporting being Francophone on representative surveys, namely, the CCHS, the NPHS, and the HHS. Many years of data were available, which increased our ability to identify Francophones for purposes of our study (that is, 1970 to 2009).

The Francophone cohort was supplemented by adding first order family members of the initial cohort. This is possible because children of Manitoba residents have the same health identification number as their parents until they are 18 years old. We estimated that about two-thirds of the additional family members would be French speaking and that the other third would be closely linked to the Francophone community through family ties. The Francophone cohort contained 46 954 people after the family linking was completed and 40 600 remained in the province and alive for the study period from 1998 to 2008. The 2006 Canadian Census estimates that there were 50 000 Francophones in Manitoba.

Permission was granted to link the Repository data to Francophones found in the CCHS (1.1, 1.2, 2.1, 2.2, 3.1, 2007, and 2008; *n* = 1627), the NPHS (1996 to 1997; *n* = 547), and the Manitoba HHS (1989 to 1990; *n* = 168). The linkage is done through a health numeric identifier, anonymously scrambled by the health department, common to the Repository data and the data collected through the surveys. A total of 2342 Francophones and 39 906 non-Francophones were available using respondents from these representative surveys. Variables (both directly reported and derived) available in these surveys included the respondent’s mother tongue, language used at home, and the first official language learned. Data from 7 CCHS cycles were pooled based on methodology recommended by Statistics Canada.^15^

Two methods were used to address possible selection biases in the Francophone and matched cohort derived from the Repository, which included matching and comparing results with a representative survey sample. Each person in the Francophone cohort (*n* = 40 600) was matched with 3 non-Francophone people (*n* = 121 800). The non-Francophones for the matching procedure were randomly pulled from a pool of all Manitobans with the same age, sex, and health districts where they lived on December 31, 1998. The health districts, determined by postal codes and used as a proxy for socioeconomic and environmental influences, have populations on average of 55 000 people. In rural areas or areas with higher concentrations of Francophones, it was necessary to search for matches in neighbouring districts. To minimize the introduction of systemic biases from the matching procedure, we used person-years to account for the varying lengths of time that the data were available for each person, such as new births and people newly eligible for health coverage. We corrected for the issue of immortal time by requiring that the non-Francophone matches be alive on the day their matched Francophone cohort member was surveyed or otherwise identified in the sample. Second, results obtained in the Repository study population were compared with results from the representative survey study population for all the mental health indicators except suicide attempts and suicide deaths.

### Data Analysis

Once both study populations were defined, they were linked at an individual level through a scrambled identifier to mental health indicators created from the Repository databases. The following database files of the Repository were accessed to create the mental health indicators: records of hospital discharges, visits to physicians outside of those occurring in hospitals, the time a person is registered as a resident of Manitoba, as well as their age, sex, area of residence, vital statistics (records of births and deaths, causes of death), pharmaceutical prescriptions dispensed, CCHS, NPHS, HHS, and census files (socioeconomic information and counts of Francophones and others at the neighbourhood level from 1990, 1996, 2001, and 2006). The definitions of these mental health variables are found in Table 1. Note that the self-reported mental health indicator was only available in the survey population data and was added to understand the perspective of Francophones regarding their mental health in a representative sample.

All data analyses were conducted using SAS version 9.3 (SAS Institute Inc, Cary, NC). RRs, in addition to crude and adjusted rates, were calculated to reflect the relative difference between both study populations. The count of events for each indicator was modelled using generalized linear modelling, suitable for nonnormally distributed data such as counts. Suicide and suicide attempts were modelled using a Poisson distribution, which is indicated for very rare events, and the other mental disorder indicators were modelled using a negative binomial distribution, which is indicated for relatively rare but highly variable counts. Age and sex were included in the models to adjust for differences in regional age and sex distributions. Self-rated mental health was directly standardized to the combined weighted CCHS survey Manitoba population aged 12 years and older. Statistical testing, using bootstrapping, was conducted to determine differences in the RRs between the Repository and survey study populations.

Logistic regression was used to explore the impact of being Francophone on positive self-rated mental health and SUD while controlling the effects of other explanatory variables from the survey data (self-rated health, *n* = 898; SUD, *n* = 1182). These in-depth analyses were conducted on these indicators to further examine unexpected differences found. Two models were examined, a basic model with adjustments for age, sex, and geographic region that closely replicated the results of our main analyses and a full model that also included sociodemographic and lifestyles variables.

## Results

Age, sex, and area-level income of the Francophones (*n* = 40 600) and the matched cohort of other Manitobans (*n* = 121 800) in the Repository study population were similar because of the matching procedure. Francophones and non-Francophones had the same percentage of males (48%) and the same age distribution (0 to 19 years: 27.4%; 20 to 64 years: 62.1%; 65 years and older: 10.5%). Overall, Francophones in rural areas are living in slightly more socioeconomically advantaged areas, and in urban areas are living in slightly less socioeconomically advantaged areas, compared with other Manitobans. The characteristics shown in Table 2 were only available in the survey study population and were used to control for the relation between being Francophone and mental health indicators. Differences were observed in the sociodemographic and lifestyle characteristics between Francophones (*n* = 1182) and non-Francophones (*n* = 18 832) in the survey study Repository.

The Repository study population in Table 3 was divided into residents from the capital city of Winnipeg and residents living outside of Winnipeg. Compared with other Manitobans, Francophones had lower rates of suicide and suicide attempts, as well as lower rates of receiving a diagnosis for SUD in rural regions outside of Winnipeg, but had similar rates on these indicators within Winnipeg. No significant differences were found in the rates of having any mental disorders, mood disorders, anxiety disorders, and dementia between the linguistic groups, both provincially and regionally.

In the survey study population shown in Table 4, no differences were found in rates of diagnosed mental disorders between Francophones and other Manitobans. Rates of diagnosed SUD in the survey sample showed a trend toward being lower for Francophones, however, this difference was not statistically significant. We compared and tested the results from Tables 3 and 4 and no differences were found between the results of both study populations. Although there appeared to be differences in the RRs, these differences were not statistically significant. This increases our confidence that results found in our study reflect the actual differences in diagnosed mental disorders between Francophones and other Manitobans.

When asked about their mental health in the CCHS, Francophones were less likely than other Manitobans to report their mental health as excellent, very good, or good (66.9%, compared with 74.2%). The first part of Table 5 shows that the results of the basic model are consistent with the results in the initial analysis in Table 4: Francophones are less likely to report positive mental health (OR 0.66; 95% CI 0.50 to 0.87). In the full model, the effect of being Francophone on mental health is accentuated (OR 0.63; 95% CI 0.47 to 0.84). This suggests that being Francophone, or having other characteristics associated with being Francophone, is associated with poorer self-rated mental health. Thus sociodemographic and lifestyle factors that were included in the model did not account for this finding.

In Table 5, we explored, in a similar way, whether the differences found in rates of diagnosis of SUD would be influenced by the addition of other sociodemographic and lifestyle factors available in the survey data. The results of the basic model are consistent with the results in Table 3: Francophones had lower rates of a diagnosis for SUD but this difference was not statistically significant (OR 0.89; 95% CI 0.57 to 1.40). In the full model, the effect of being Francophone on diagnosis of SUD is accentuated (OR 0.84; 95% CI 0.76 to 0.93) and is statistically significant. This suggests that after controlling for sociodemographic and lifestyle factors being Francophone is associated with lower rates of diagnosed SUD.

## Discussion

To our knowledge, the mental health of Francophones in Manitoba has not been examined using administrative databases or representative survey data. We had expected to find no differences in the mental health indicators between the linguistic groups because we had carefully selected a matched cohort for comparison by age, sex, and geographic region as a proxy for SES. The finding that no differences were found in the rates of mood disorders, anxiety, and dementia are consistent with more recent studies.^7^,^16^ Lower rates of suicide attempts and suicide deaths were found among Francophones living outside of Winnipeg. Earlier studies showed that Francophones in Canada had higher suicidality than Anglophones^4^,^5^; however, the study by Clarke et al^5^ also noted that these differences were explained by inequalities in SES and a poor sense of community belonging. According to Statistics Canada, Francophones in Manitoba have comparable income and education levels with other Manitobans.^17^ Manitoba is also one of the few provinces where French and English have official language status in the provincial courts and legislative assembly. Francophones have their own school division, a French-language university, access to some French-language health services, and a rich cultural life, all of which potentially contributes to a sense of belonging.

Some of the social and cultural protective factors surrounding suicidality may account for the lower rates of diagnosed SUD among Francophones living outside of Winnipeg. Further analyses with the survey data confirmed that being Francophone was associated with lower SUD rates after controlling for a range of sociodemographic and lifestyle factors. Alcohol use in social settings is well accepted in French culture as it is among Francophones in Manitoba, which could potentially be both a risk factor and a protective factor. European research found that binge drinking is more prevalent in Northern, Western, and Eastern countries than those in the southern parts of Europe, suggesting that “binge drinking was less likely in countries in which alcohol is integrated into everyday life compared to countries where heavy drinking on week-ends was more culturally accepted.”^18^^, p 123^

The apparent contradiction found in our study between the self-rated mental health survey item and the mental illness diagnoses recorded in the administrative data may be because we are measuring 2 different aspects of mental health. Responses to this self-rated question were closely associated with responses from the Mental Health Continuum, an extensive measure of emotional, psychological, and social well-being.^19^ Keyes19 contends that mental health is a broader construct than the absence of mental illness and that other dimensions of mental health should be considered in determining the mental health of a population.^19^ It has been argued that living in a linguistic minority may be negatively impacting this population’s well-being.^20^ Recent research has found that cultural identity factors are associated with positive self-esteem and psychological well-being.^21^ Living in a linguistic minority context may be influencing broader dimensions of mental health without having a negative impact of rates of diagnosis on mental disorders.

In our study, the mental health indicators, with the exception of the self-rated mental health indicator, were calculated for residents who were diagnosed by a physician, and therefore were among those who actually sought help. Francophones living in an environment where French is a minority language have limited access to French-language health services, and accordingly may not be seeking help for mental health concerns. This may partly explain the discrepancy between the poorer self-rated mental health and lower or similar rates of diagnosed mental disorders. Research has shown that the positive impact of language in health care delivery is through clear communication with the health care practitioners and through its role in the therapeutic relationship. People are more likely to express their concerns, ask questions, and follow health recommendations if they are well connected to their health care providers.^22^ According to a report entitled Minorities Speak Up,^23^ 61% of French-speaking Manitobans considered it important to have health services in French, but only 14% reported communicating with their family doctor in French.

Studying the health of Francophones in Canada has been challenging because of the lack of population-based databases with the language variables required to identify them.^24^ The Repository of administrative data allowed us to create a cohort of Francophones and a matched cohort of non-Francophones. Many years of data are included in the Repository, so that patients could be selected at many points in time. We are confident that patients in the Francophone cohort are Francophones or are closely related to them through family ties. A selection bias could be present as not all were identified with the use of administrative databases because some are not requesting French services or attending French-speaking facilities. Francophones who were not included may be different culturally and health-wise than those who were not. Comparing results from the Francophone and matched cohort and the survey data provided assurance that this selection bias was not a strong factor.

The matching technique used also strengthens our confidence in the results found in our study. Ensuring that each Francophone selected was matched with 3 other Manitobans on age, sex, and geographic region provided a method for controlling for these important factors. We were able to ensure that the area-level indicator of SES was comparable across groups. We felt that this was a reasonable comparison as Francophones in Manitoba are not socioeconomically disadvantaged, but rather are similar to other Manitobans regarding income, education, and employment levels.

Rates of diagnosed mental disorders of Francophones and non-Francophones were based on actual medical claims, hospitalizations, and prescriptions. The strength of these indicators is that they provide objective rates of people treated for mental disorders and are not biased by self-assessment or recall. While they have been carefully created through consultation with health professionals, an important limitation is that they have not been validated by comparing to a gold standard, such as a standardized interview by a mental health specialist. Another limitation is that we cannot ascertain whether the differences are because help seeking behaviours may differ between linguistic populations. A recent report documented differences in diagnosed rates of mood disorders and SUD across regions in Manitoba using these indicators.^25^ Suicide and suicide attempts, based on Vital Statistics as well as health data, are less prone to help seeking biases because of their severity.

## Conclusion

Our study shows that Francophones in Manitoba have similar rates of diagnosed mental disorders as other Manitobans, with the exception of Francophones outside the large urban area of Winnipeg who have lower rates of diagnosed SUD and lower rates of suicide attempts and deaths. However, Francophones were less likely to rate their mental health as positive, compared with other Manitobans. This discrepancy in how Francophones view their mental health and their rates of diagnosed mental health problems may be related to health seeking behaviours in the Francophone population. Community and government agencies should try to improve the mental health of this population through mental health promotion and by addressing language and cultural barriers to health services. Further research can consider if there are differences in health care seeking behaviour that may be affecting the findings presented here. Also of interest would be studying whether policies developed within Manitoba regarding linguistic rights contribute to the mental health status of Francophones.

## Figures and Tables

**Table 1:** Definitions of mental health indicators

Indicator	Definition
Suicide and suicide attempts	The proportion of Manitoba residents who completed or attempted suicide. Age- and sex-adjusted annual prevalence of suicide or suicide attempts for residents age 10 and older was measured for calendar years 1999 to 2007. Suicides were defined as any death record in Vital Statistics data, with self-inflicted injury or poisoning listed as the primary cause of death. Suicide attempts were defined as hospitalization for suicide and self-inflicted injury or accidental poisoning, with a consultation to psychiatry.
Any diagnosed mental disorder	The percentage of residents age 10 and older who received treatment for 1 or more of the following mental illnesses: mood disorders, anxiety disorders, SUD, personality disorder, and schizophrenia. The age- and sex-adjusted prevalence of the mental illness disorders was measured for residents aged 10 and older in fiscal years 2004/05 to 2008/09. Personality disorder was defined as 1 or more hospitalizations with a diagnosis for a personality disorder (ICD-9-CM code 301; ICD-10-CA codes F34.0, F60, F61, F62, F68.1, F68.8, and F69) or 1 or more physician visits with a diagnosis for a personality disorder (ICD-9-CM code 301). Schizophrenia was defined as 1 or more hospitalizations with a diagnosis for schizophrenia (ICD-9-CM code 295; ICD-10-CA codes F20, F21, F23.2, and F25) or 1 or more physician visits with a diagnosis for schizophrenia (ICD-9-CM code 295). The definitions for SUD, mood disorders, anxiety disorders, and dementia are below.
SUD	The percentage of residents age 10 and older diagnosed with SUD over a 5-year period (2004/05 to 2008/09) by either: One or more hospitalizations with a diagnosis for alcoholic or drug psychoses, alcohol or drug dependence, or nondependent abuse of drugs (ICD-9-CM codes 291, 292, 303, 304, and 305; ICD-10-CA codes F10-F19 and F55) or One or more physician visits with a diagnosis for alcoholic or drug psychoses, alcohol or drug dependence, or nondependent abuse of drugs (ICD-9-CM codes 291, 292, 303, 304, and 305).
Anxiety disorder	The percentage of residents age 10 and older diagnosed with an anxiety disorder over a 5-year period (2004/05 to 2008/09) by any 1 of the following conditions: One or more hospitalizations with a diagnosis for anxiety states, phobic disorders, or obsessive–compulsive disorders (ICD-9-CM codes 300.0, 300.2, and 300.3; ICD-10-CA codes F40, F41.0, F41.1, F41.3, F41.8, F41.9, and F42) or Three or more physician visits with a diagnosis for anxiety disorders (ICD-9-CM code 300).
Dementia	The percentage of residents age 55 and older diagnosed with dementia in a 5-year period (2004/05 to 2008/09) by any 1 of the following conditions: One or more hospitalizations with a diagnosis for dementia, including organic psychotic conditions, cerebral degenerations, and senility (ICD-9-CM codes 290, 291.1, 292.2, 292.82, 294, 331, and 797; ICD-10-CA codes F00, F01, F02, F03, F04, F05.1, F06.5, F06.6, F06.8, F06.9, F09, F10.7, F11.7, F12.7, F13.7, F14.7, F1.57, F16.7, F18.7, F19.7, G30, G31.0, G31.1, G31.9, G32.8, G91, G93.7, G94, and R54) or One or more physician visits with a diagnosis for dementia (ICD-9-CM codes 290, 294, 331, and 797).
Mood disorders	The percentage of residents age 10 and older diagnosed with mood disorders over a 5-year period by any 1 of the following conditions: One or more hospitalizations with a diagnosis for depressive disorder, affective psychoses, neurotic depression, or adjustment reaction (ICD-9-CM codes 296.2–296.8, 300.4, 309, and 311; ICD-10-CA codes F31, F32, F33, F341, F38.0, F38.1, F41.2, F43.1, F43.2, F43.8, F53.0, and F93.0) or One or more physician visits with a diagnosis for depressive disorder, affective psychoses, or adjustment reaction (ICD-9-CM codes 296, 309, and 311) or One or more hospitalizations with a diagnosis for anxiety disorders (ICD-9-CM code 300; ICD-10-CA codes F32.0, F34.1, F40, F41, F42, F44, F45.0, F451, F452, F48, F68.0, and F99) and 1 or more prescriptions for an AD or mood stabilizer (ATC codes N03AB02, N03AB52, N03AF01, N05AN01, and N06A) or One or more physician visits with a diagnosis for anxiety disorders (ICD-9-CM code 300) and 1 or more prescriptions for an AD or mood stabilizer (N03AB02, N03AB52, N03AF01, N05AN01, and N06A)
Self-rated mental health	The adjusted and weighted proportion of survey respondents aged 12 and older who answered positively to the question in the CCHS cycles 2.1, 2.2, 3.1, 2007, and 2008, “In general, would you say your mental health is: (excellent, very good, good, fair, or poor)?” This indicator was calculated by taking the ratio of the number of respondents who rated their health as excellent, very good, or good to the number of all respondents.

AD = antidepressant; ATC = anatomical therapeutic chemical; CCHS = Canadian Community Health Survey; ICD-9-CM = International Classification of Diseases, Ninth Revsion with Clinical Modifications; ICD-10-CA = International Classification of Diseases, Tenth Revsion with Canadian Enhancements; SUD = substance use disorder

**Table 2:** Sociodemographic characteristics of the Francophones and all other Manitoba survey respondents in the survey data study population

Covariate	Francophone weighted *n* = 1182 % (95% CI)^a^	All other Manitobans weighted *n* = 18 832 % (95% CI)^a^
Sex		
Male	45.35 (39.95–50.76)	49.44 (48.63–50.25)
Female	54.65 (49.24–60.06)	50.56 (49.75–51.37)
Geographical region		
Rural south	32.73 (27.54–37.91)	18.99 (18.12–19.86)
Mid	6.70 (4.75–8.66)	13.54 (12.77–14.31)
North	1.74 (1.11–2.38)^b^	3.55 (3.29–3.80)
Brandon	1.67 (1.00–2.34)^b^	4.45 (4.13–4.77)
Winnipeg	57.16 (51.45–62.87)	59.47 (58.37–60.58)
Marital status		
Married or common law	67.05 (52.42–71.69)	65.55 (64.36–66.74)
Single	32.95 (28.32–37.58)	34.45 (33.26–35.65)
High school education		
Graduate	72.90 (68.45–77.35)	76.75 (75.73–77.77)
Did not graduate	27.10 (22.65–31.55)	23.25 (22.24–24.27)
Employment status		
Currently employed	63.89 (58.98–68.79)	68.81 (67.83–69.80)
Unemployed	36.11 (31.21–41.02)	31.19 (30.20–32.18)
Sense of belonging to local community		
Yes	67.37 (62.19–72.55)	65.22 (63.69–66.75)
No	32.63 (27.45–37.81)	34.78 (33.25–36.31)
Smoking status		
Currently smoking	28.44 (21.35–35.53)	25.96 (24.44–27.47)
Nonsmoker	71.56 (64.47–78.65)	74.04 (72.53–75.56)
Leisure time physical activity		
Active	23.51 (18.80–28.22)	20.74 (19.74–21.73)
Moderate	27.50 (22.17–32.83)	24.16 (23.09–25.24)
Inactive	48.99 (43.13–54.86)	55.10 (53.77–56.43)
Fruit and vegetable consumption		
≥5 times per day	34.01 (28.09–39.94)	32.24 (30.23–34.24)
<5 times per day	65.99 (60.06–71.91)	67.76 (65.76–69.77)
Continuous covariate	Weighted mean (95% CI)^a^	Weighted mean (95% CI)^a^

Age, years	48.36 (46.86–50.08)	45.37 (45.08–45.64)
Household income, Can$	50 110 (46 670–53 110)	51 180 (50 350–51 860)
Body mass index, kg/m^2^	27.38 (26.90–27.83)	27.49 (27.35–27.63)

a Confidence intervals were calculated using bootstrapping.

b The estimated weighted per cent is highly variable and should be interpreted with caution.

**Table 3:** RRs of mental health indicators for the population-based Repository study population

Indicator	Francophone adjusted % (95% CI)	Other Manitobans adjusted % (95% CI)	Rate ratio (95% CI)	χ^2^	*df*	*P*
Suicide and suicide attempts						
Manitoba	49.33 (40.61–59.92)	70.85 (64.78–76.94)	0.70 (0.57–0.85)	13.32	1	<0.001
Winnipeg	51.54 (38.13–69.69)	59.63 (50.77–70.04)	0.91 (0.69–1.19)	0.48	1	0.49
Outside Winnipeg	42.30 (30.58–58.52)	72.47 (62.37–84.21)	0.59 (0.43–0.79)	12.25	1	<0.001
Any diagnosed mental disorder						
Manitoba	23.80 (21.23–26.68)	24.33 (24.06–24.60)	0.98 (0.87–1.10)	0.21	1	0.65
Winnipeg	25.85 (22.65–29.50)	25.97 (22.89–29.46)	1.00 (0.87–1.13)	0.08	1	0.77
Outside Winnipeg	21.31 (18.90–24.03)	21.91 (19.55–24.55)	0.97 (0.87–1.08)	0.26	1	0.61
Diagnosed substance use disorder						
Manitoba	3.66 (3.13–4.28)	4.36 (4.23–4.49)	0.84 (0.72–0.98)	5.07	1	0.02
Winnipeg	4.03 (3.37–4.80)	4.61 (3.97–5.35)	0.87 (0.74–1.04)	3.33	1	0.07
Outside Winnipeg	3.18 (2.64–3.83)	3.96 (3.38–4.63)	0.80 (0.68–0.95)	6.51	1	0.01
Diagnosed depression						
Manitoba	19.42 (17.27–21.85)	19.46 (19.21–19.71)	1.00 (0.89–1.12)	0.01	1	0.91
Winnipeg	21.19 (18.51–24.27)	20.87 (18.35–23.74)	1.02 (0.89–1.16)	0.11	1	0.74
Outside Winnipeg	17.41 (15.40–19.70)	17.33 (15.45–19.50)	1.00 (0.90–1.12)	0.01	1	0.94
Diagnosed anxiety disorder						
Manitoba	7.85 (6.87–8.97)	7.96 (7.78–8.13)	0.99 (0.86–1.13)	0.02	1	0.89
Winnipeg	8.38 (7.17–9.79)	8.82 (7.65–10.17)	0.95 (0.81–1.11)	0.53	1	0.47
Outside Winnipeg	7.17 (6.17–8.34)	6.77 (5.90–7.76)	1.06 (0.92–1.21)	0.67	1	0.41
Diagnosed dementia						
Manitoba	16.61 (14.20–19.43)	15.97 (15.44–16.49)	1.04 (0.89–1.22)	0.19	1	0.66
Winnipeg	17.45 (15.87–19.18)	18.12 (17.18–19.11)	0.96 (0.89–1.05)	1.31	1	0.25
Outside Winnipeg	12.72 (11.16–14.49)	11.96 (11.06–12.94)	1.06 (0.93–1.22)	0.81	1	0.37

**Table 4:** RRs of mental health indicators for the representative survey study population

Indicator^a^	Francophone weighted adjusted % (95% CI)	Other Manitobans weighted adjusted % (95% CI)	Rate ratio % (95% CI)	*Z*	*P*
Self-rated mental health	66.88 (62.05–71.71)	74.23 (73.13–75.34)	0.90 (0.83–0.97)	−2.9073	0.004
Any diagnosed mental disorder	25.39 (22.20–28.59)	23.40 (22.61–24.18)	1.09 (0.95–1.22)	1.2092	0.23
Diagnosed substance use disorder	3.43 (1.95–4.92)^b^	3.90 (3.52–4.28)	0.88 (0.49–1.27)	−0.6021	0.55
Diagnosed mood disorder	21.00 (18.09–23.92)	18.64 (17.96–19.32)	1.13 (0.97–1.29)	1.5724	0.12
Diagnosed anxiety disorder	7.22 (5.53–8.91)	7.55 (7.06–8.04)	0.96 (0.72–1.19)	−0.3695	0.71
Diagnosed dementia	9.44 (6.37–12.51)^b^	7.61 (6.94–8.28)	1.24 (0.82–1.66)	1.1331	0.26

a The estimates in suicide and suicide attempts were suppressed owing to small numbers.

b The estimated weighted rate is highly variable and should be interpreted with caution.

**Table 5:** Weighted logistic regression models predicting self-rated mental health and a diagnosis of substance use disorder, survey data study population

Covariate	Self-rated mental health weighted adjusted OR (95% CI^a^)		Substance abuse diagnosis weighted adjusted OR (95% CI^a^)	
	Basic model	Full model	Basic model	Full model
Francophone cohort (compared with all other survey respondents)	0.66 (0.50–0.87)^b^	0.63 (0.47–0.84)^b^	0.89 (0.57–1.40)	0.84 (0.76–0.93)^b^
Age	0.99 (0.99–1.00)^b^	1.00 (0.99–1.00)^b^	0.99 (0.98–1.00)^b^	1.00 (0.99–1.00)^b^
Males (compared with females)	1.02 (0.90–1.16)	0.98 (0.85–1.14)	1.72 (1.33–2.24)^b^	1.71 (1.63–1.79)^b^
Geographical regions (compared with Winnipeg)				
Rural south	0.91 (0.79–1.06)	0.96 (0.81–1.13)	0.82 (0.60–1.13)	0.83 (0.78–0.88)^b^
Mid	0.79 (0.68–0.92)^b^	0.80 (0.68–0.93)^b^	1.07 (0.74–1.56)	1.02 (0.95–1.10)
North	0.97 (0.77–1.22)	1.05 (0.82–1.34)	2.05 (1.51–2.79)^b^	1.73 (1.62–1.84)^b^
Brandon	0.81 (0.63–1.03)	0.82 (0.64–1.04)	1.15 (0.70–1.90)	1.14 (1.08–1.21)^b^
Married or common law (compared with single)		1.22 (1.04–1.43)^b^		0.89 (0.84–0.94)^b^
Household income (per $10 000)		1.01 (1.00–1.01)^b^		1.00 (1.00–1.00)^b^
High school graduate (compared with not)		1.50 (1.26–1.79)^b^		0.93 (0.89–0.97)^b^
Currently employed (compared with not)		1.18 (1.00–1.40)^b^		0.81 (0.77–0.86)^b^
Sense of belonging to local community (compared with no)		1.94 (1.68–2.24)^b^		0.78 (0.65–0.95)^b^
≥5 drinks on 1 occasion, once per month or more (compared with <once per month)		0.94 (0.77–1.15)		
Current smoker (compared with not)		0.76 (0.66–0.88)^b^		5.72 (4.93–6.64)^b^
Body mass index		0.99 (0.98–1.00)^b^		1.01 (1.00–1.01)^b^
Leisure time Physical Activity Index (compared with inactive)				
Active		1.65 (1.38–1.98)^b^		0.71 (0.65–0.79)^b^
Moderate		1.42 (1.22–1.65)^b^		0.52 (0.50–0.55)^b^
Consumes fruits and (or) vegetables ≥5 times a day (compared with <5)				0.84 (0.85–1.22)

a Confidence intervals were calculated using bootstrapping.

b Statistically significant at *P* < 0.05

## Abbreviations

CCHS: Canadian Community Health Survey
HHS: Heart Health Survey
MCHP: Manitoba Centre for Health Policy
NPHS: National Population Health Survey
RR: rate ratio
SES: socioeconomic status
SUD: substance use disorder
